# Genetic ancestry and population differences in levels of inflammatory cytokines in women: Role for evolutionary selection and environmental factors

**DOI:** 10.1371/journal.pgen.1007368

**Published:** 2018-06-07

**Authors:** Song Yao, Chi-Chen Hong, Edward A. Ruiz-Narváez, Sharon S. Evans, Qianqian Zhu, Beverly A. Schaefer, Li Yan, Marie V. Coignet, Kathryn L. Lunetta, Lara E. Sucheston-Campbell, Kelvin Lee, Elisa V. Bandera, Melissa A. Troester, Lynn Rosenberg, Julie R. Palmer, Andrew F. Olshan, Christine B. Ambrosone

**Affiliations:** 1 Department of Cancer Prevention and Control, Roswell Park Comprehensive Cancer Center, Buffalo, NY, United States of America; 2 Department of Nutritional Sciences, University of Michigan School of Public Health, Ann Arbor, MI, United States of America; 3 Department of Immunology, Roswell Park Comprehensive Cancer Center, Buffalo, NY, United States of America; 4 Department of Biostatistics and Bioinformatics, Roswell Park Comprehensive Cancer Center, Buffalo, NY, United States of America; 5 Department of Pediatric Hematology & Oncology, Roswell Park Comprehensive Cancer Center, Buffalo, NY, United States of America; 6 Department of Pediatric Hematology & Oncology, Jacobs School of Medicine and Biomedical Sciences, University of Buffalo, Buffalo, NY, United States of America; 7 Department of Biostatistics, Boston University School of Public Health, Boston, MA, United States of America; 8 College of Pharmacy, The Ohio State University, Columbus, OH,United States of America; 9 Cancer Prevention and Control Program, Rutgers Cancer Institute of New Jersey, The State University of New Jersey, New Brunswick, NJ, United States of America; 10 Department of Epidemiology, Gillings School of Global Public Health, University of North Carolina at Chapel Hill, Chapel Hill, NC, United States of America; 11 Slone Epidemiology Center at Boston University, Boston, MA, United States of America; University of Washington, UNITED STATES

## Abstract

Selection pressure due to exposure to infectious pathogens endemic to Africa may explain distinct genetic variations in immune response genes. However, the impact of those genetic variations on human immunity remains understudied, especially within the context of modern lifestyles and living environments, which are drastically different from early humans in sub Saharan Africa. There are few data on population differences in constitutional immune environment, where genetic ancestry and environment are likely two primary sources of variation. In a study integrating genetic, molecular and epidemiologic data, we examined population differences in plasma levels of 14 cytokines involved in innate and adaptive immunity, including those implicated in chronic inflammation, and possible contributing factors to such differences, in 914 AA and 855 EA women. We observed significant differences in 7 cytokines, including higher plasma levels of CCL2, CCL11, IL4 and IL10 in EAs and higher levels of IL1RA and IFNα2 in AAs. Analyses of a wide range of demographic and lifestyle factors showed significant impact, with age, education level, obesity, smoking, and alcohol intake, accounting for some, but not all, observed population differences for the cytokines examined. Levels of two pro-inflammatory chemokines, CCL2 and CCL11, were strongly associated with percent of African ancestry among AAs. Through admixture mapping, the signal was pinpointed to local ancestry at 1q23, with fine-mapping analysis refined to the Duffy-null allele of rs2814778. In AA women, this variant was a major determinant of systemic levels of CCL2 (p = 1.1e-58) and CCL11 (p = 2.2e-110), accounting for 19% and 40% of the phenotypic variance, respectively. Our data reveal strong ancestral footprints in inflammatory chemokine regulation. The Duffy-null allele may indicate a loss of the buffering function for chemokine levels. The substantial immune differences by ancestry may have broad implications to health disparities between AA and EA populations.

## Author summary

Individuals of European and African ancestry have different susceptibility for developing specific infections and diseases. Part of this difference in immune response is thought to arise from genetic differences accumulated over the millennia that conferred advantages in fighting different infectious pathogens endemic to different parts of the world. The impact of these immune differences and how they are influenced by modern lifestyles and living environments remains to be understood. Findings from this study revealed population differences in the levels of circulating cytokines, i.e. chemical messengers of the immune system, which were due in part to different demographic and lifestyle factors. Further, a change in the gene encoding for the Duffy antigen receptor protein, identified as rs2814778 and known as the Duffy-null allele, was the most important factor explaining low circulating levels of CCL2 and CCL11, key chemokines regulating the migration of white blood cells, specifically monocytes and eosinophils, which play a role in inflammation. This genetic variant occurs almost exclusively among Africans, likely because of its role in protecting against malaria infection, and results in loss of Duffy antigen protein on red blood cells. The substantial immune differences by ancestry may have broad implications for health disparities.

## Introduction

The human immune system provides a primary defense against pathogens external to and within the body. After the “Out of Africa” diaspora approximately 60,000 years ago, human ancestors encountered vastly different pathogenic environments, and their survival and reproductive fitness depended on how successful their immune systems fought off infections without the aid of modern medicine. It has been hypothesized that strong selection pressure due to exposures to life-threatening infectious pathogens endemic to Africa, particularly malaria, shaped a pro-inflammatory immune milieu in populations of African ancestry (AA). This is supported by evidence from evolutionary genetics [1–3], and data showing that genomic regions hosting immunity-related genes were under stronger selection pressure than the rest of the human genome [4]. We and others have shown that AAs have a higher frequency of variants related to pro-inflammatory cytokines but a lower frequency of variants related to anti-inflammatory cytokines [5–8]. Many variants associated with infectious, autoimmune, and inflammatory diseases discovered from genome-wide association studies (GWAS) display extreme differences in allele frequencies across populations [9]. These ancestral genetic variations that were shaped by human evolutionary history likely remain influential on the constitutive immune milieu in populations today.

The environment that the immune system interacts with is likely another determinant of immune variations, and for most of the developed world, differs greatly from that experienced by early humankind. Infectious and other pathogens are no longer a common threat to most of the world populations; whereas longer life expectancy, over-nutrition, sedentary lifestyle, and socioeconomic stress are part of a macroenvironment that can have a profound impact on the immune system. A twins study found that many immune parameters became more divergent between monozygotic twins with increasing age, highlighting the importance of environmental factors in driving immune variations [10]. Population differences in exposures, in concert with genetic make-up, likely account for a large portion of immune variations across populations.

Three recent studies demonstrated marked population differences in transcriptional responses of *in vitro* cultured T-cells, monocytes and macrophages under stimulated conditions [11–13]; yet few have examined population differences in constitutional immune state under unstimulated conditions. Even less is known regarding genetic and environmental contributions to such differences. Here, we report findings from a comprehensive study integrating genetic and epidemiologic data with measures of 14 circulating cytokines, to investigate differences in immune parameters between AA and European Ancestry (EA) women, and genetic and environmental contributions to those differences.

## Results

### Population differences in plasma levels of cytokines between AA and EA women

A total of 914 AA and 855 EA women were included in the study. Selected descriptive characteristics of the study population are summarized by study and race in **S1 Table**. The average age was 52 years in both AAs and EAs and approximately 40% were premenopausal; nevertheless, there were significant population differences in body mass index (BMI), waist-to-hip ratio (WHR), family history of breast cancer, education attainment, cigarette smoking, alcohol drinking, and developmental and reproductive history.

**Fig 1:**
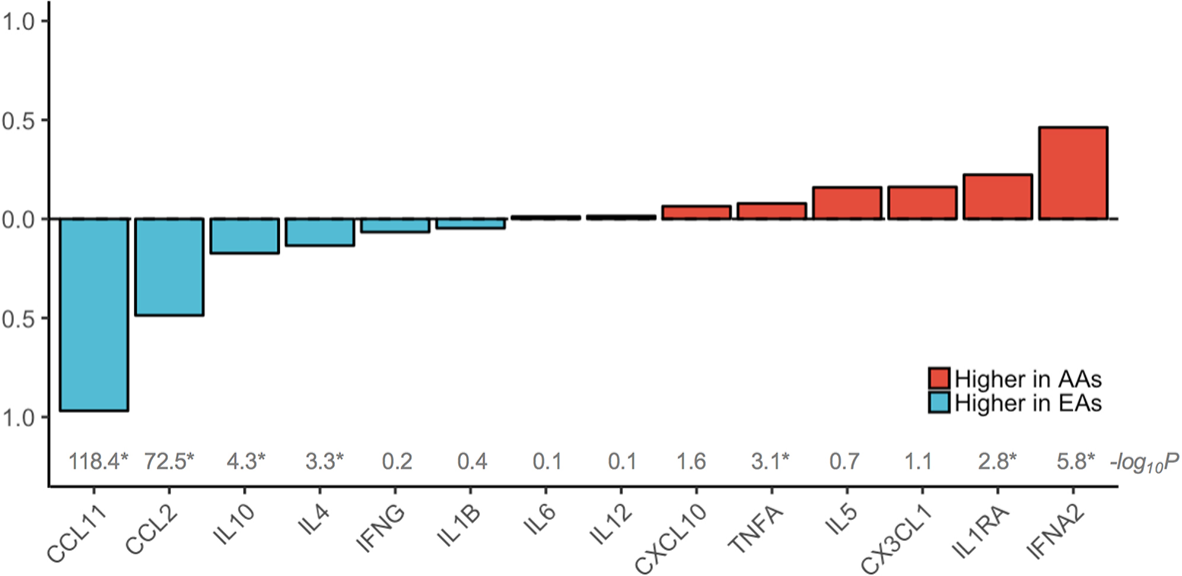
Population differences in plasma levels of cytokines. Population differences in plasma levels of the 14 cytokines were calculated as the percent difference between the subgroup means within African American (AA) and European American (EA) women divided by the overall mean. The means were calculated with transformed levels, and the percent differences were calculated after data back-transformation. Cytokines with a higher level in EAs are in blue and those with a higher level in AAs are in red. P-values from t-test by population are shown as negated log10-transformed values, with the asterisks indicating markers with a significant population difference with a p-value <3.6e-3 after Bonferroni correction for testing of 14 markers. The population differences remained significant after controlling for technical variables including study, season and year of blood collection.

Of 14 cytokines analyzed, half showed significant population differences. As shown in **Fig 1**, the mean concentrations of CCL11, CCL2, IL10 and IL4 were significantly higher in EAs (97%, 49%, 17% and 13% higher, respectively), while the mean concentrations of IFNα2, IL1RA and TNFα were significantly higher in AAs (46%, 23% and 8% higher, respectively). These differences remained statistically significant after correcting for multiple comparisons, as well as controlling for technical variables, and were also confirmed when the data were analyzed as categorical variables in quartiles. No differences were found for the other 7 cytokines, including IFNγ, IL1β, IL5, IL6, IL12, CXCL10, CX3CL1, after correction for multiple comparisons.

### Environmental factors and plasma cytokines levels

**Fig 2:**
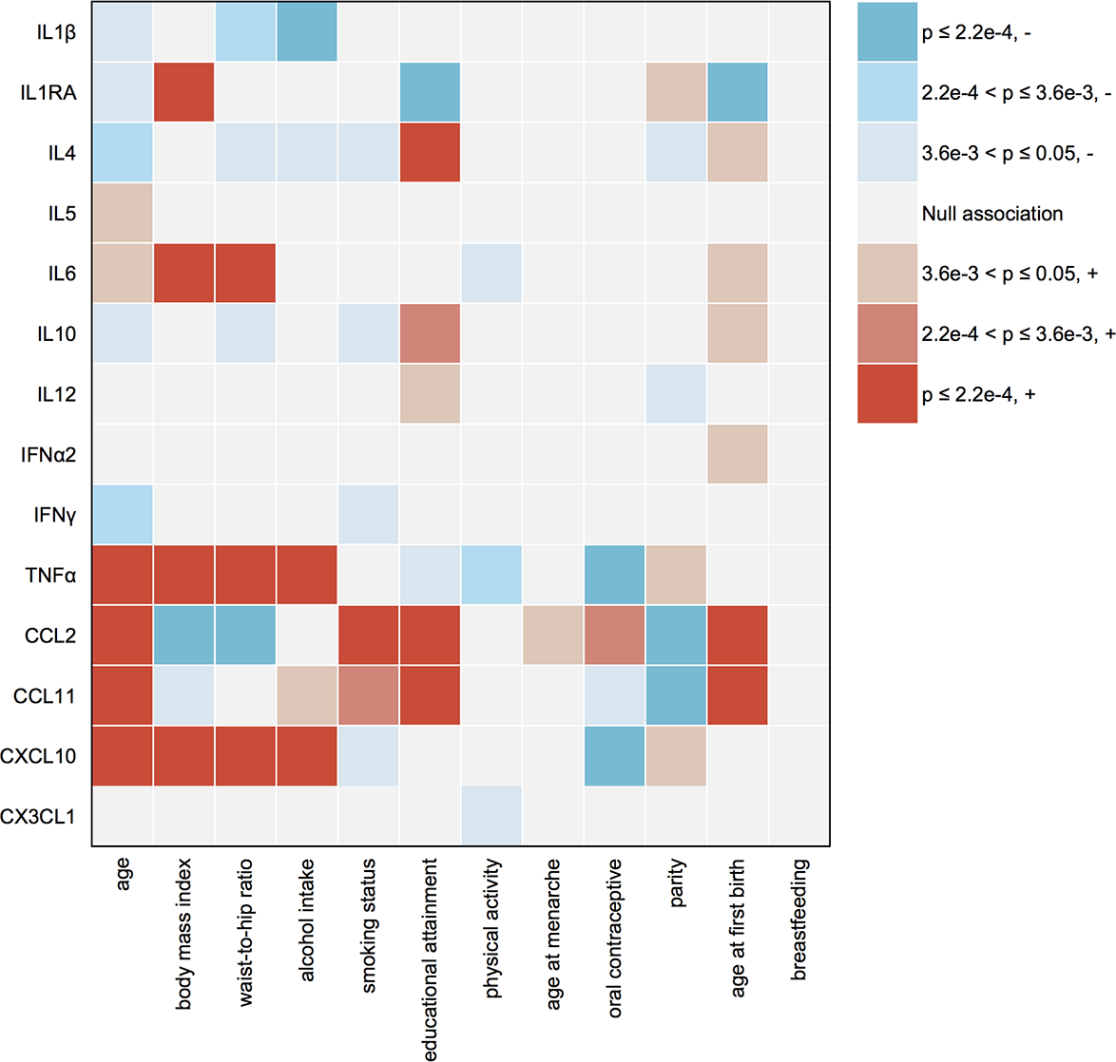
Table plot of associations between cytokine levels and epidemiologic factors. Plasma levels of the 14 cytokines were tested in relation to each epidemiologic factor using linear regression models, with and without adjustment for covariates including study, age, season and year of blood collection, race, and body mass index (BMI), except for situations when a covariate itself is the main factor of interest (age and BMI). The adjusted and unadjusted results are consistent, and thus the p-values from unadjusted models are used for this plot. For age at first birth and breastfeeding, the analyses are restricted to parous women. Type I error rates of 0.05, 0.0036 and 0.00022 are used to indicate no correction for multiple testing, correction for 14 markers, and correction for 14 markers multiplied by 16 epidemiologic factors, respectively. The P-value for each association is color coded as illustrated by the legends. The direction of an association is determined by trend test treating the epidemiologic factor as an ordinal variable. “-” means an environmental factor is associated with decreased marker level, and “+” means the opposite.

A number of highly significant associations were observed between environmental factors and cytokine levels, mostly among pro-inflammatory cytokines (**Fig 2**; summary statistics of all the association testing are available in **S2 Table**). The results were consistent with and without adjustment for covariates, including study, age, season and year of blood collection, race, and BMI, except for situations when age or BMI was the main factor of interest. Many of the relationships were in the expected direction. These include older age with higher levels of pro-inflammatory IL6, TNFα, CCL2, CCL11 and CX3CL1, but lower levels of anti-inflammatory IL4 and IL10; higher obesity (measured by body mass index and waist-to-hip ratio) with higher levels of IL6, TNFα, CXCL10, and IL1RA as a marker of the activity of pro-inflammatory IL1; alcohol intake with higher levels of TNFα and CXCL10; smoking with higher levels of CCL2 and CCL11; and higher physical activity with lower levels of IL6 and TNFα. Some of the relationships were novel, including alcohol intake with higher levels of pro-inflammatory IL1β, and educational attainment as a crude proxy of socioeconomic status with several cytokines that showed no clear preference towards either pro- or anti-inflammation. In addition, reproductive history also appeared to have an impact on plasma cytokines. Oral contraceptive use and parity were associated with lower levels of pro-inflammatory cytokines, whereas late age at first birth was associated with higher levels of pro-inflammatory cytokines. These epidemiologic associations were consistent between AA and EA women in stratified analyses.

**Fig 3:**
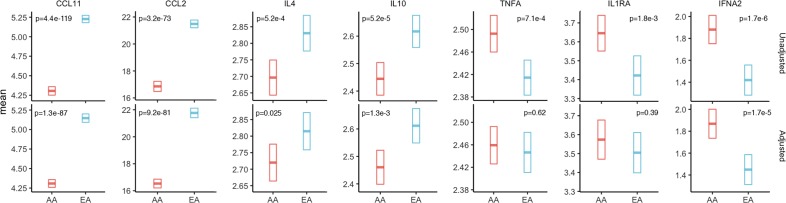
Unadjusted and adjusted means of cytokine concentrations by population. Seven cytokines that were significantly different between African American (AA) and European American (EA) women were further tested in linear regression models with adjustment for study, season and year of blood collection, and in addition, a set of environmental factors that were associated with each analyte in previous analyses. These include: age, body mass index (BMI), waist-hip-ratio (WHR), education, smoking, age at menarche, oral contraceptive use, parity, and age at first childbirth for CCL2; age, BMI, education, alcohol drinking, smoking, oral contraceptive use, parity, and age at first childbirth for CCL11; age, WHR, education, alcohol drinking, smoking, parity, and age at first childbirth for IL4; age, WHR, education, smoking, and age at first childbirth for IL10; age, BMI, education, parity, and age at first childbirth for IL1RA; age, BMI, WHR, education, alcohol drinking, physical activity, oral contraceptive use, and parity for TNFα; and age at first childbirth for IFNα2. The bars in the middle of the boxes indicate the unadjusted mean and adjusted least square means, with the lower and upper edge of the boxes corresponding to the lower and upper 95% confidence interval for the means and least square means. The levels were natural log-transformed for all markers except CCL2 which was square root-transformed.

Of the 7 cytokines showing significant population differences, the significance for CCL2, CCL11, IL10 and IFNα2 remained after adjusting for all the technical and epidemiological factors identified in the above analyses and controlling for multiple comparisons (**Fig 3**). For TNFα and IL4, the primary contributing factor to population differences was higher obesity in AA women and higher education in EA women, respectively; whereas for IL1RA, obesity, education, and age at first childbirth were contributing factors to observed differences.

### Mapping of genetic determinants of CCL2 and CCL11 levels in AA women

**Fig 4:**
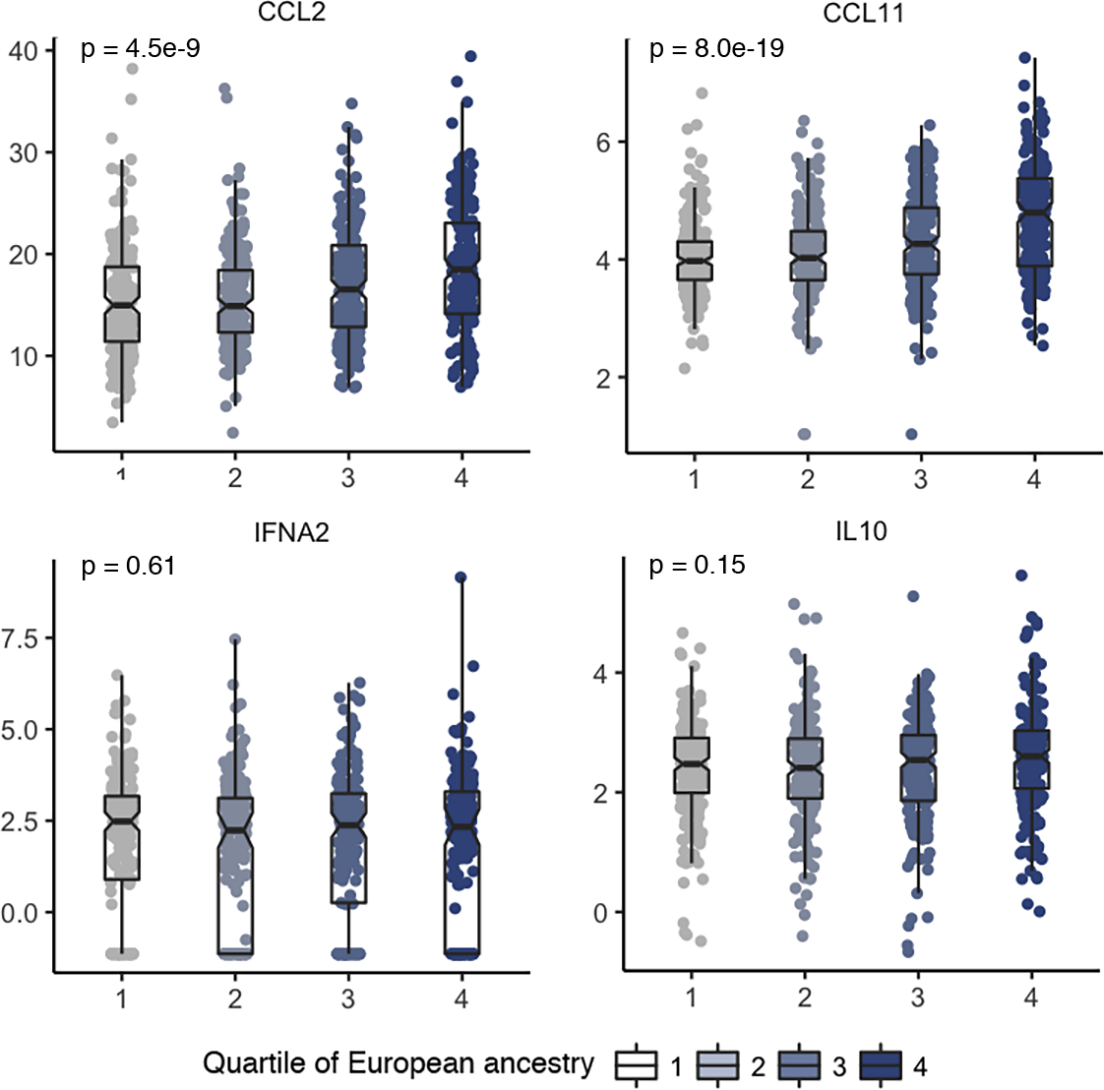
Concentrations of cytokines by quartiles of the estimated global genetic ancestry in African American women. Plasma concentrations of the four cytokines that remained significantly different between African American (AA) and European American (EA) women after controlling for covariates are plotted by the quartiles of the estimated global genetic ancestry among AA women. The quartiles for the estimated European genetic ancestry are: ≤0.08 (n = 200), 0.09–0.12 (n = 198), 0.13–0.20 (n = 201), and ≥0.21 (n = 201). Each dot is a sample and the samples are grouped by quartiles of genetic ancestry. The bar in the middle of a notched box indicates the subgroup median, and the lower and upper edges indicate the first and third quartiles, respectively. The concentrations were natural log-transformed for all markers except CCL2 which was squared root-transformed. The p-values were from an ANOVA test, which remained essentially unchanged after controlling for covariates including study, season and year of blood collection, age and body mass index (BMI) in linear regression models.

**Fig 5:**
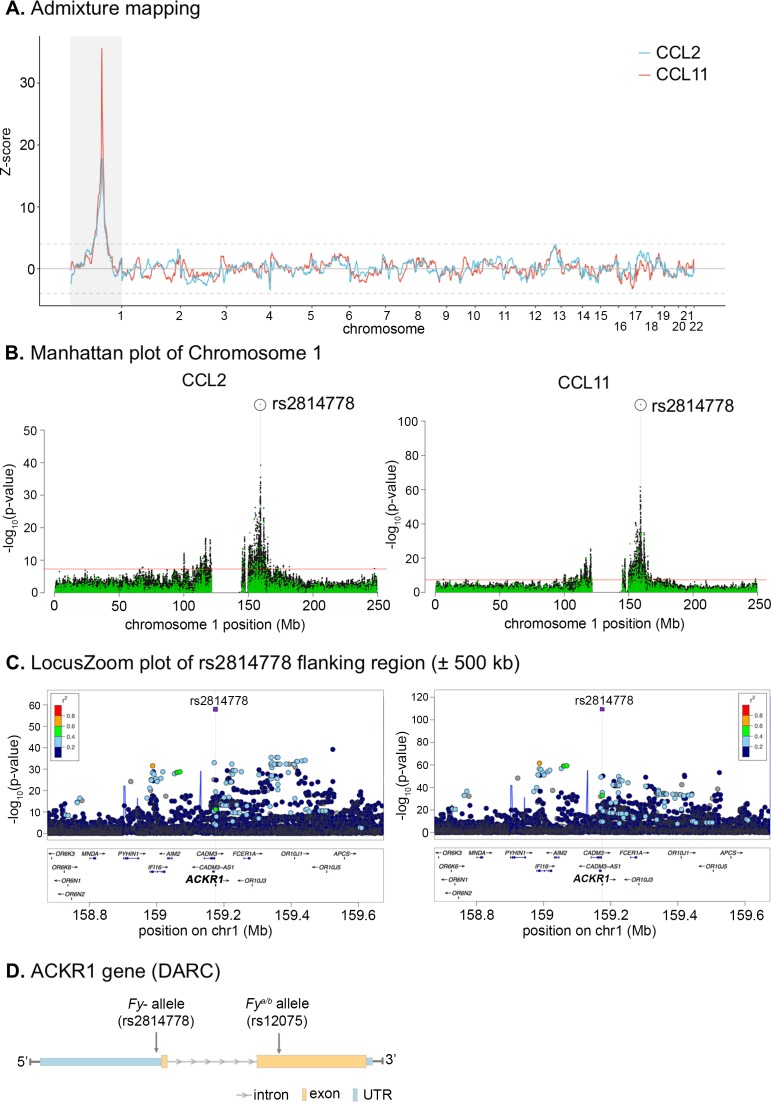
Mapping of genetic determinants of CCL2 and CCL11 in African American women. Admixture mapping and fine-mapping analyses identify the Duffy null (*FY*-*null*) variant (rs2814778) as the lead variant for lower plasma concentrations of CCL2 and CCL11 in African American (AA) women. **A.** Z-score from admixture mapping analyses of plasma CCL11 and CCL2 using 2,624 ancestry informative markers selected from the exome genotyping array. The reference lines are Z-score = -4 or 4, which are deemed genome-wide significant. Positive Z-score indicates excess European local ancestry and negative Z-score indicates deficit European local ancestry. The models were adjusted for study, season and year of blood collection, global ancestry estimates, and epidemiological factors associated with each marker. Blue line is for CCL2 and red line is for CCl11. The shaded area covers chromosome 1 and is the region subjected for fine-mapping analysis in panel B. **B.** Manhattan plots of variants in chromosome 1 from linear regression analysis of plasma CCL2 and CCL11. Green dots are typed variants and black dots are imputed variants. Linear regression models are adjusted for study, season and year of blood collection time, and top 5 principal components (PCs) for population structures. Additional adjustment for epidemiological factors associated with each marker did not substantially change the results. The top hit rs2814778 is circled. The shaded area is a 1 mega-base flanking region centered on the top hit which is further depicted using LocusZoom in panel C. **C.** LocusZoom plots for a 1 mega-base flanking region centered on the top hit rs2814778 for plasma CCL2 and CCL11. Squares are typed variants and dots are imputed variants. The r^2^ estimate used for color coding the linkage disequilibrium between each marker and rs2814778 is taken from 1000 Genome data from populations of African descent. Linear regression models are adjusted for study, year and season of blood collection time, and top 5 principal components (PCs) for population structures. Additional adjustment for epidemiological factors associated with each marker did not substantially change the results. The shaded area covers the genetic region of *ACKR1* gene depicted in panel D. **D.** Schema of *ACKR1* gene (also known as *DARC*). The gene has a very short exon and a longer exon, separated by only one intron. The top hit rs2814778 (*FY-null* allele) locates in the 5’ untranslated region (UTR) which contains a GATA3 transcription factor binding site. Another functional variant rs12075 (FY^*^A/FY^*^B) locates in the long exon and results in missense amino acid change.

In further analyses of the four cytokines with the estimated global genetic ancestry among 809 AA women from whom dense genotyping data were available, the levels of CCL2 and CCL11, but not IFNα2 or IL10, were significantly inversely associated with the percent of European ancestry (**Fig 4**). Based on the strong evidence of ancestral influence on CCL2 and CCL11 levels, admixture mapping analysis was performed with adjustment for global ancestry, study, season and year of blood collection, and significant epidemiological factors for each marker were adjusted for in the analysis. **Fig 5A** shows a very strong signal of excess European ancestry on chromosome 1 with an identical peak for CCL2 and CCL11. This region spans approximately 83 megabase encompassing the centromere, with a central peak in 1q23.2 tagged by rs6678328 (Z-score = 17.8, p = 3.2e-71 for CCL2 and Z-score = 35.6 and p = 7.2e-278 for CCL11). *CCL2* and *CCL11* are two paralog genes and both reside in a chemokine gene cluster on 17q11.2–12.

**Fig 6:**
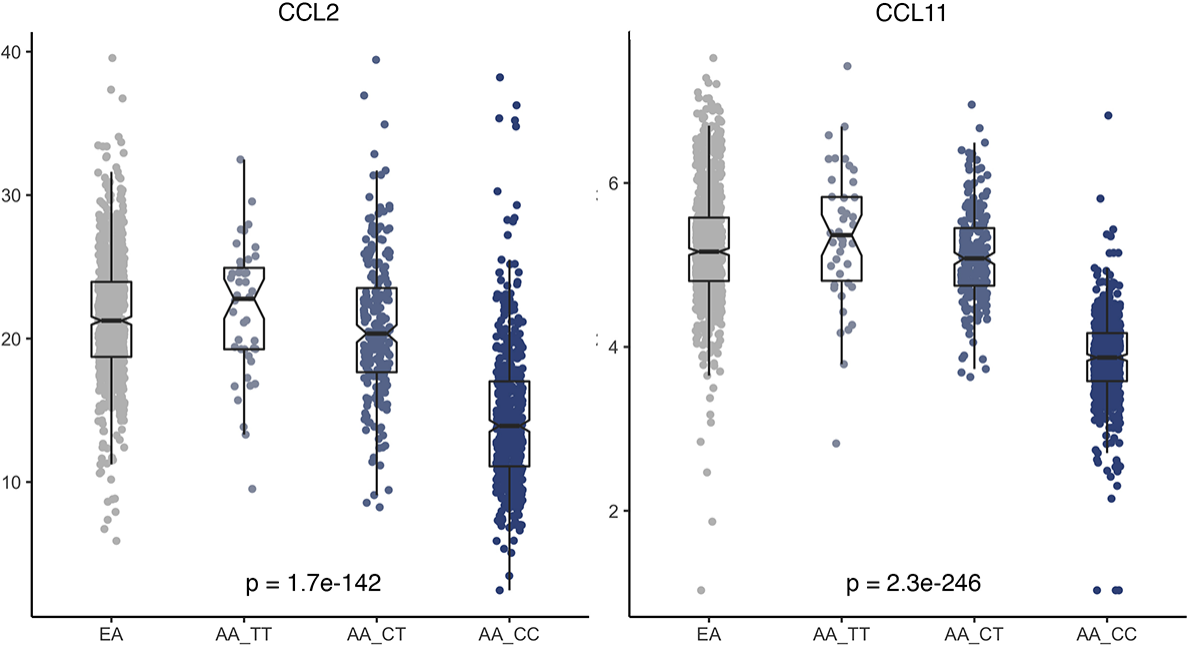
Plasma concentrations of CCL2 and CCL11 by population and rs2814778 genotype. Plasma concentrations of the CCL2 and CCL11 by population (European Americans, or EAs, are presumably to be all TT genotype according to ExAC data) and rs2814778 genotype (TT, CT and CC) in African Americans. Each dot represents a sample, and the bar in the middle of a notched box indicates the subgroup median, and the lower and upper edges indicate the first and third quartiles, respectively. The extended lines indicate the range in each subgroup. The data were squared root-transformed for CCL2 and natural log-transformed for CCL11. The p-values were from ANOVA test across the four groups, which remained essentially unchanged after controlling for covariates including study, season and year of blood collection, age and body mass index in linear regression models.

In fine-mapping analysis focusing on chromosome 1 with adjustment for study, season and year of blood collection, the top 5 principal components (PCs), age, and significant epidemiological factors identified with each chemokine, the top hit was a genotyped variant rs2814778 for both CCL2 (p = 1.1e-58) and CCL11 (p = 2.2e-110) (**Fig 5B**). The associations with rs2814778 were consistent between WCHS and CBCS samples (**S3 Table**) and across the quartiles of percent of European ancestry (**S4 Table**). Plasma levels of CCL2 and CCL11 were lowest among AA women carrying the CC genotype of this variant (66%), compared to AA women carrying TT (5%) or CT genotypes (29%), or to EA women (assumed to be nearly 100% TT genotype according to ExAC data [14]), consistent with a recessive model of the C allele (**Fig 6**). In likelihood ratio test comparing different genetic models for rs2814778, as expected, the recessive model out performed the dominant or additive model. In conditional analysis adjusting for rs2814778, none of the other variants on chromosome 1 remained significant for either CCL2 or CCL11 (p>5e-8 for all variants tested). In multivariate analysis, it became clear that rs2814778 drives the admixture mapping signal on chromosome 1 as well as the associations with global genetic ancestry (**S5 Table**). This variant alone was estimated to explain 19% and 40% of variance in the levels of CCL2 and CCL11, respectively, among AA women.

The top hit rs2814778 on chromosome 1 locates in 1q23.2, the peak region identified in admixture mapping. More specifically, it resides in the 5’ untranslated region of atypical chemokine receptor 1 (*ACKR1*), which is also known as Duffy antigen/chemokine receptor (*DARC*) (**Fig 5C**). The T to C transition, also referred to as *FY-null* allele, results in the disruption of a GATA motif (**Fig 5D**) and subsequently the loss of erythrocyte cell surface expression of Duffy blood group antigen, a mechanism to resist erythrocyte invasion by the human malaria parasites *Plasmodium vivax* and *P*. *Knowles* into red blood cells [15]. Erythrocyte DARC also binds to chemokines including CCL2 and CCL11, serving as a reservoir buffering blood chemokine concentrations [16]. The T to C transition was associated with markedly lower concentrations of CCL2 and CCL11 among AA women in our study. Another functional exonic single nucleotide polymorphism (SNP) rs12075 located in the longer exon of the gene results in an amino acid change (Gly44Asp), and determines which of the FY^*^A and FY^*^B Duffy alleles are expressed, if any (**Fig 5D**). In previous GWAS of CCL2 concentrations in EAs and Hispanics, rs12075 was identified as the top hit [17–19]. This SNP was associated with CCL2 (p = 6.3e-12) and CCL11 (p = 9.8e-34) concentrations in our AA population. The association of rs12075 with CCL2 became suggestive when conditioning on rs2814778 (p = 5.2e-5) or when the analysis was restricted to Duffy-positive AAs (p = 0.001); nevertheless, the association of rs21075 with CCL11 became non-significant under both conditions (p = 0.23 and p = 0.61, respectively).

## Discussion

This epidemiologic study began with a survey of population differences in systemic levels of cytokines. The initial findings led to investigation of a comprehensive list of environmental factors and further, genetic variants at a genome-wide scale, as potential contributors to such differences. The major findings are three-fold.

First, there were significant population differences in half of the 14 cytokines examined. Although AA women had moderately higher levels of pro-inflammatory Th-1 cytokine TNFα and lower levels of anti-inflammatory Th-2 cytokines IL4 and IL10 than EA women; there were no population differences in several widely-studied pro-inflammatory cytokines including IL1, IL6, IL12, or IFNγ. To the contrary, EA women had markedly higher levels of pro-inflammatory chemokines CCL2 and CCL11, but lower levels of type I interferon IFNα2, a prominent antiviral cytokine and also an early response cytokine that helps the transition of the immune system from an innate to an adaptive response and promotion of Th-1 cell development. Based on these comparisons, it may be prudent to conclude that systemic immune environment in AAs is not necessarily more pro-inflammatory than in EAs, as there are likely significant interpersonal variations, depending on specific immune components, concomitant comorbid conditions, and environmental stimuli. Our findings, along with the previous in vitro studies of immune cell transcriptomic response [11–13], demonstrate that ancestry is a major contributor to immune variations across populations.

Second, environmental factors were important determinants of circulating cytokine levels. We provide herein a comprehensive account of a range of demographic, anthropometric, socioeconomic, lifestyle and reproductive history factors in relation to systemic immune markers, which has long been speculated but lacking in support by high-quality data [20, 21]. Notably, those associations were consistent when examined separately within AA and EA groups, with no apparent evidence of modifying effects by ancestry, indicating that the observed population differences in cytokines may be, in part, due to disproportionate exposure to those environmental factors. Indeed, after those factors were controlled for, population differences in concentrations of IL4, TNFα and IL1RA diminished.

Third, there is a strong shared genetic basis for population differences in circulating levels of CCL2 and CCL11. This signal is essentially identical to those from previous admixture mapping analyses of white blood cell count, neutrophil count and multiple sclerosis susceptibility among AAs [22–24]. The lead variant for white blood cell count and neutrophil count was ultimately mapped to rs2814778 in *DARC* [24–26], the well-known *FY-null* allele that is almost fixed in West and Central Africa and very rare or non-existent in populations elsewhere [27–29]. It is widely speculated that the geographic diversity of this allele was a result of strong positive selection for protection from malaria infection endemic to West Africa thousands of years ago [27, 28, 30]. It would have been impossible to identify this signal if the analysis were conducted among populations of non-African ancestry. Indeed, in GWAS analysis of circulating CCL2 levels among European and Hispanic populations, another *DARC* variant, rs12075 that determines the FY^*^A and FY^*^B Duffy alleles, was identified instead [17–19]. In our study, rs12075 was also associated with chemokine levels in AA women; yet the associations depended mainly on rs2814778. Although previous hypothesis-driven studies have noted lower levels of CCL2 among Duffy negative individuals [31, 32], our study, using an agnostic approach, provides the strongest evidence, to date, of the dominant effects of *FY-null* allele in determining the levels of those two chemokines. However, it should be noted that the Illumina exome array has a relatively sparse coverage of non-exonic regions. Although imputation was performed to increase marker density and low frequency and poorly imputed SNPs were removed from the analysis, additional independent signals may be identified using denser SNP arrays or through sequencing.

DARC is an atypical chemokine receptor on erythrocytes that binds with high-affinity to a large number of CXC and CC inflammatory chemokines, including CCL2 and CCL11 [33]. It functions as chemokine “sink” to absorb and remove excessive chemokines from local microenvironment of inflammatory sites [34], as well as a “reservoir” to release chemokines when their concentrations are low in circulation [35]. Thus, erythrocytic DARC may work as a buffering system to regulate the homeostasis of chemokine levels by storing them in red blood cells and providing a constant chemokine source to replenish their circulating levels during continued chemokine extraction from blood in liver [16]. When this buffering function is lost among Duffy-null individuals, at unstimulated conditions at the basal state as the situation in our study, CCL2 and CCL11 may undergo a faster removal for lack of buffering. Our results are consistent with early studies in animals and humans [31, 32, 36], which support a positive correlation between erythrocytic DARC and systemic levels of chemokines CCL2 and CC11.

Therefore, the *FY-null* allele may cause disruption to a fine-balanced uniform level of chemokine responsiveness, rendering a hypersensitive chemokine signaling. In a mouse study, those receiving DARC-negative erythrocytes had increased neutrophil infiltration into the lungs, increased levels of inflammatory cytokines, and increased lung microvascular permeability, in comparison to those receiving wildtype DARC erythrocytes [37]. In humans, Duffy-null individuals were more sensitive to CCL2-induced monocyte mobilization [31]. It was also reported that AA patients who were DARC negative had lower allograft survival after kidney transplant than DARC-positive patients, possibly due to less controlled inflammatory responses [38].

It is possible that changes to the immune system brought by Duffy negativity simply define benign physiological variations, such as “benign ethnic neutropenia” [39]. Given the versatile role that immunity plays in a myriad of human diseases, it is also possible that those changes bear health significance, especially for chronic diseases with a later age of onset. The absence of Duffy antigen has been linked to a number of conditions, from neutropenia to complications of sickle cell anemia, transplant rejection, and psoriasis [16]. However, data are sparse on the associations of Duffy null genotype with cancer, for which immune dysregulation is a hallmark. Experimental studies support DARC as a negative regulator of tumor growth by inhibiting tumor angiogenesis and metastasis via scavenging angiogenetic chemokines [40, 41]. Its expression on endothelial cells also inhibited metastasis through interaction with CD82/KAI on tumor cells to induce their senescence [42]. Nevertheless, it should be noted that the function of DARC is tissue specific. The Duffy null genotype appears to affect only erythrocytes, with little impact on other tissues. Thus, its link with cancers, if any, would be mediated through chemokine regulation by erythrocytes. Indeed, CCL2 and CCL11 have been implicated in several cancers, including breast cancer etiology and metastasis [43–45]. Because of the striking difference in the prevalence of the *FY-null* allele between AA and EA populations, it will also be interesting to study whether this ancestry rooted genetic marker is biologically implicated in ethnic disparities in immunity-related diseases and conditions [46, 47].

In conclusion, our study demonstrated significant population differences in systemic levels of inflammatory cytokines between AA and EA women, attributable to both environmental and genetic factors. We identified the Duffy-null allele as a major determinant of the lower levels of pro-inflammatory chemokines CCL2 and CCL11 in AA women, suggesting that the ancestral genetic trait selected to protect from malaria infection thousands of years ago continues to have an influence on human immunity at a population level. Whether the resulting changes represent benign variations or have an impact on human health warrants future investigation.

## Methods

### Ethics statement

This research was approved by the Institutional Boards of Roswell Park Cancer Institute(#I-177810 approval date 6/28/17 for one year), Rutgers Cancer Institute of New Jersey (#02-2011-0240, no expiration), and University of North Carolina Chapel Hill (#11–1277 approval date 1/18/18 for one year). In both studies, patients completed the informed consent process during the in-person interview.

### Study populations

Data and biospecimens were drawn from two case-control studies in the African American Breast Cancer Etiology and Risk (AMBER) Consortium [48] that had blood samples available from controls: the Women’s Circle of Health Study (WCHS) and the Carolina Breast Cancer Study (CBCS). WCHS is a population-based case-control study first established in 2002 in the New York City (NYC) metropolitan area [49, 50]. Controls were identified through random digital dialing. Blood samples were collected at enrollment in the first five years of the study. The CBCS is a population-based case-control study in North Carolina (NC) first established in 1993 [51]. Controls were identified through Division of Motor Vehicle lists and Health Care Finance Administration lists. Blood samples were collected at enrollment in Phases 1 and 2 of CBCS. For both WCHS and CBCS, only blood samples from women enrolled as controls were included in this study. The research was approved by the Institutional Review Boards (IRB) of all participating institutes.

For both WCHS and CBCS, epidemiologic data were collected through interviewer-administered questionnaires at the time of enrollment, and anthropometric measurements were collected at the same time. Centralized data harmonization was performed in the context of AMBER to reconcile and derive common variables [48]. A total of 1,769 women (914 AAs and 855 EAs) enrolled as controls in the WCHS and the CBCS were included in the analysis of plasma cytokines.

### Measurements of plasma cytokines and chemokines

Luminex Multi-Analyte Profiling (xMAP) immune-bead array assays were used to measure plasma levels of a panel of 16 cytokines (IFNγ, IFNα2, TNFα, IL1β, IL1RA, IL4, IL5, IL6, IL10, IL12p40, IL12p70, CCL2, CCL7, CCL11, CXCL10, CX3CL1) in two multiplexes. One multiplex high sensitivity kit was used for IL1β, IL4, IL6 and IL10, (Millipore, HSCYTMAG-60SK); the remaining cytokines were measured using regular kits (Millipore, HCYTOMAG-60 K). Assays were performed in a 96-well plate format with experimental samples tested in duplicates. For quality control purposes, 5% blinded duplicates, standards, and internal quality control (QC) samples were included in each plate. Analyte capture was carried out according to manufacturer’s instructions. Data were acquired using Luminex 100 with xPONENT version 3.1 software, and concentrations measured using BeadView Analysis Software. Plate-specific nine-point standard curves were generated using the “Best Fit” curve fitting routine which automatically selects the best curve algorithm for each analyte. The samples were processed by Roswell Park Comprehensive Cancer Center Data Bank and Biorepository (DBBR), and the Luminex assays performed by Roswell Park Flow Cytometry Shared Resource using a Luminex 100 instrument.

The mean of duplicate pairs was taken as the final concentration of each sample, and outliers defined as 2.5 times interquartile range outside the first and third quartiles were removed. For values below the lower detection limit, single imputation was performed using half of the lower detection limit; and for values above the upper detection limit, similar imputation was performed using the upper detection limit value. Two analytes, IL12p40 and CCL7, were below the lower detectable limit in more than two thirds of the samples and were thus excluded from further analysis. QC indices and summary statistics for the 14 analytes are shown in **S6 Table**. Because different anti-coagulants were used by WCHS (heparin) and CBCS (citrate), and heparin is known to release erythroid CCL2 into plasma [17, 52], the levels of CCL2 and CCL11 were compared between the two studies. CCL2 levels were indeed lower in CBCS than in WCHS, whereas CCL11 levels were higher in CBCS. Thus, study was adjusted as a covariate in all analyses, and stratified analyses by study were performed when necessary.

### Genotyping and ancestry informative markers (AIMs)

Genotyping methods of the AMBER Consortium have been described in detail in previous publications [53–56]. Genotyping assays were performed by the Center for Inherited Disease Research using the Illumina Human Exome Beadchip v1.1 array. Custom content was added to boost coverage for regions of high interest, which had a total of 246,519 single nucleotide polymorphism (SNPs) including 433 genes in 45 curated pathways in innate and adaptive immune response. Data QC and imputation to the 1000 Genome Project reference data were performed by University of Washington Center for Biomedical Statistics. Marker-level filters applied were GenCall (GC) score <0.15, poor cluster properties, call rate <0.98, Hardy-Weinberg Equilibrium P <1e-4, >1 Medelian error in HapMap trios, >2 discordant calls in duplicate samples, and mitochondrial and Y chromosome SNPs, all of which resulted in the removal of 14,814 SNPs (6%). A total of 6,936 samples were genotyped and approximately 1.6% with a call rate <98% were removed. Crypt relatedness (n = 270) and outlying individuals from principal component analysis (n = 35) were flagged for sensitivity analysis. Imputation to the 1000 Genome Project data was performed using IMPUTE2 program [57] and any SNPs with a minor allele frequency (MAF) <0.01 or imputation info score <0.5 were removed. Because only AA women were included in the AMBER genotyping project, for the present analysis, a total of 809 AA healthy controls from WCHS and CBCS who also had plasma cytokines data were included for genotype analysis. As part of the standard content of the exome array, data on a total of 2,624 autosomal AIMs were available for global ancestry estimation by STRUCTURE program [58] and for admixture mapping analysis by ADMIXMAP [59].

### Statistical analysis

Data of plasma cytokines were either log- or squared root-transformed to increase normality of distribution. The pairwise correlations between cytokines were moderate to strong and similar between AA and EA women in stratified analyses (**S1 Fig**). Differences in plasma cytokines between AA and EA women were evaluated by t-tests and multivariable linear regression with adjustment for technical variables (study, and season and year of blood collection). To assess whether any population difference was attributable to a range of epidemiologic factors, including age, education, BMI, WHR, alcohol consumption, smoking, physical activity, and developmental and reproductive history, univariate analyses were first performed and significant factors were then included in a multivariable linear regression model for each cytokine. Least square means in the two populations were derived after controlling for the effect of epidemiologic factors.

To test whether genetic ancestry explained the population differences in cytokine levels after epidemiologic factors had been accounted for in AA women, the following three analyses were performed, all in AA women only because EA women were not included in the genotyping study. First, four markers which remained significantly different between AA and EA women were tested across the quartiles of global genetic ancestry in AA women. Second, for CCL2 and CCL11, the two chemokines associated with global genetic ancestry, admixture mapping analysis was performed to relate the two chemokine levels to the locus admixture proportion estimated based on the data of 2,624 selected autosomal AIMs using ADMIXMAP program [53]. Global individual admixture, study, season and year of blood collection, and significant epidemiological factors for each marker were adjusted for in the analysis. A threshold of |Z| > 4.0 was considered genome-wide significant, with a negative Z-score indicating a negative association with European ancestry at a locus of interest with the cytokine levels and a positive Z-score indicating a positive association. Third, fine-mapping analyses were performed using PLINK [60] on the imputed and genotyped exome array data in the dosage format for chromosome 1, where admixture mapping revealed a strong signal of local ancestry associated with both chemokines. A total of 1,278,281 SNPs on chromosome 1 with MAF ≥0.01 and info score ≥0.5 were tested in linear regression models, including 80,630 genotyped SNPs. Covariates adjusted in the multivariate linear models included study, season and year of blood collection, the top 5 PCs, age, and significant epidemiological factors identified with each chemokine. Conditional analyses on rs2814778 as the most significant variant were also performed by including the variant as an additional covariate in the linear model in an attempt to identify additional independent signals. All analyses were performed using R program unless otherwise specified, and the Bonferroni method was used to correct for multiple testing except for fine-mapping genotype analysis where p <5e-8 was used as genome-wide significance cutoff.

## Supporting information

S1 Table Descriptive characteristics of the healthy controls from the Carolina Breast Cancer Study (CBCS) and the Women’s Circle of Health Study (WCHS) with plasma samples. (XLSX)

S2 Table Associations of the 14 cytokines with epidemiologic factors. (XLSX)

S3 Table Associations of rs2814778 with CCL2 and CCL11 by study. (XLSX)

S4 Table Associations of rs2814778 with CCL2 and CCL11 by quartiles of percent European ancestry in African American women. (XLSX)

S5 Table Multivariable analysis of global genetic ancestry, local ancestry, and top hits on chromosomes 1. (XLSX)

S6 Table Summary statistics and QC indices of measured cytokines. (XLSX)

S1 Fig Correlation plots of plasma levels of cytokines. (TIF)
