# Supplementary figures and images for: Genetic ancestry and population differences in levels of inflammatory cytokines in women: Role for evolutionary selection and environmental factors
Source: PLoS Genet. 2018 Jun 7;14(6):e1007368. doi: 10.1371/journal.pgen.1007368 (PMC5991662; doi:10.1371/journal.pgen.1007368)

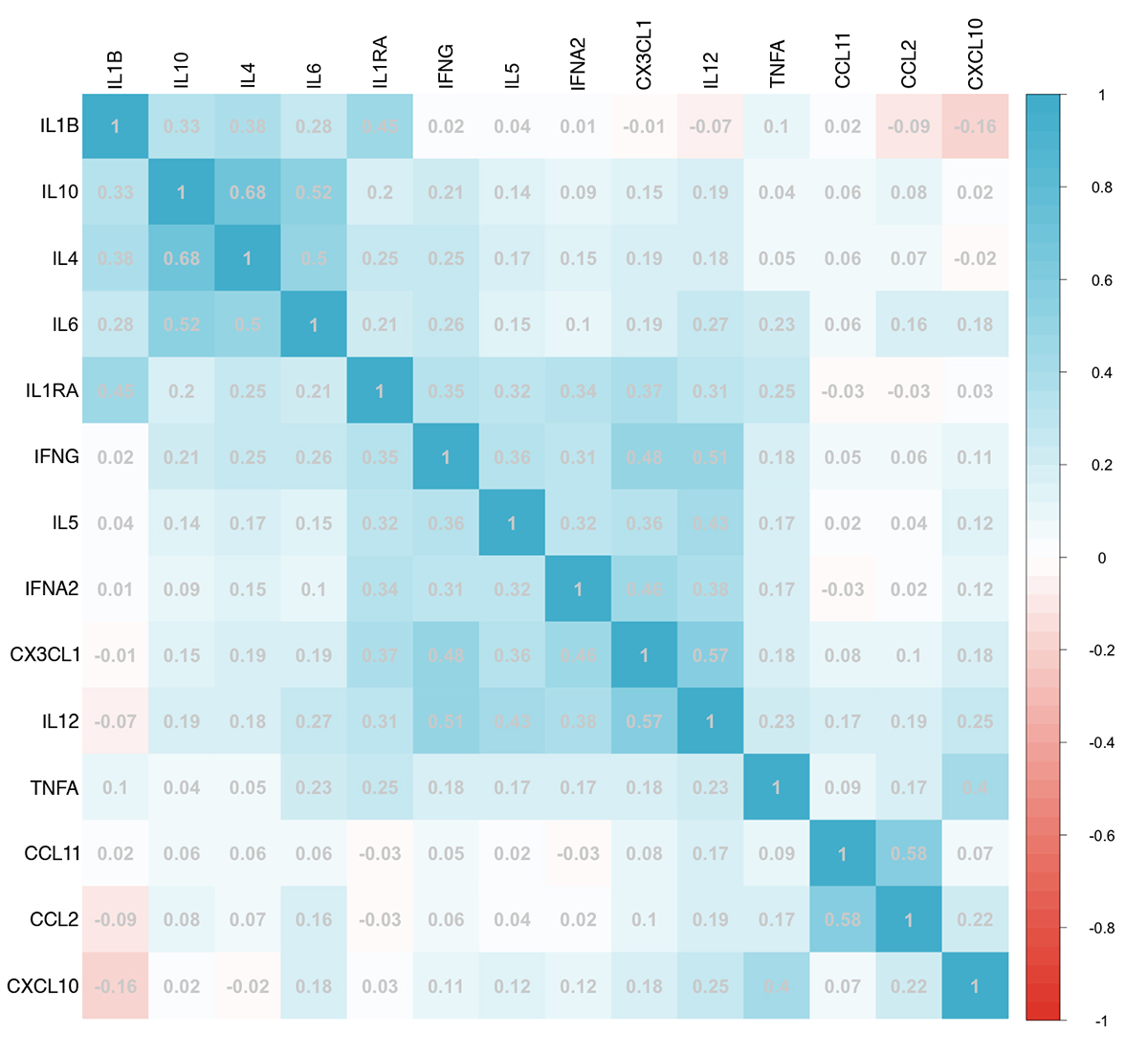

Supplement: S1 Fig — (TIF) [file pgen.1007368.s007.tif]
